# On the role and opportunities in teamwork design for advanced multi-robot search systems

**DOI:** 10.3389/frobt.2023.1089062

**Published:** 2023-04-13

**Authors:** Roee M. Francos, Alfred M. Bruckstein

**Affiliations:** Multi-Agent Robotic Systems Laboratory, Department of Computer Science, Technion- Israel Institute of Technology, Haifa, Israel

**Keywords:** multiple mobile robot systems, teamwork analysis, motion and path planning for multi agent systems, aerial robots, robot surveillance and security, collaborative algorithms for motion planning of autonomous robots

## Abstract

Intelligent robotic systems are becoming ever more present in our lives across a multitude of domains such as industry, transportation, agriculture, security, healthcare and even education. Such systems enable humans to focus on the interesting and sophisticated tasks while robots accomplish tasks that are either too tedious, routine or potentially dangerous for humans to do. Recent advances in perception technologies and accompanying hardware, mainly attributed to rapid advancements in the deep-learning ecosystem, enable the deployment of robotic systems equipped with onboard sensors as well as the computational power to perform autonomous reasoning and decision making online. While there has been significant progress in expanding the capabilities of single and multi-robot systems during the last decades across a multitude of domains and applications, there are still many promising areas for research that can advance the state of cooperative searching systems that employ multiple robots. In this article, several prospective avenues of research in teamwork cooperation with considerable potential for advancement of multi-robot search systems will be visited and discussed. In previous works we have shown that multi-agent search tasks can greatly benefit from intelligent cooperation between team members and can achieve performance close to the theoretical optimum. The techniques applied can be used in a variety of domains including planning against adversarial opponents, control of forest fires and coordinating search-and-rescue missions. The state-of-the-art on methods of multi-robot search across several selected domains of application is explained, highlighting the pros and cons of each method, providing an up-to-date view on the current state of the domains and their future challenges.

## 1 Introduction

In this paper, we provide an extensive survey of the current literature and the state-of-the-art of the field as well as our perspective and our opinion on its most intriguing future research directions based on a thorough literature survey and our experience in investigating and developing algorithms for multi-robot systems.

A cooperating team of robots offers several benefits over a single robotic platform such as reliability and robustness through redundancy, reduced time for completing the task, the possibility to operate in a larger environment, task decomposition and allocation, combined with the ability to use heterogeneous robots with different capabilities to optimally complete a designated mission. In intelligent robotics tasks, teamwork can be achieved with either direct, indirect or without any communication between the robots comprising the team, which are also referred to as agents throughout this article. Direct communication relies on the broadcast of information between agents in order to achieve the desired team’s goal. There are many forms for indirect communication such as sensing the environment or observing the behaviors of other agents in order to plan an agent’s plan. Having no communication implies that agents are oblivious to the actions of other agents in the team and operate with a plan that is irrespective of the plans of other agents in the team.

**FIGURE 1:**
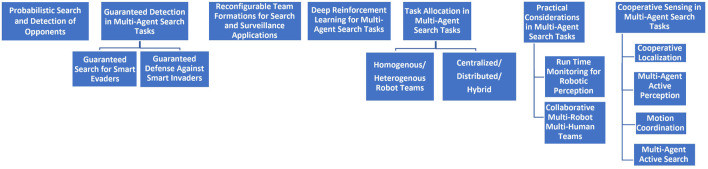
Classification of future research directions in multi-agent search tasks.

This paper highlights different challenges and aspects related to team cooperation in multi-agent search tasks. The chosen topics were distilled from recent major conferences and journal papers concerned with multi-agent search tasks. We chose to focus on specific research topics we believe are the most promising for future research. We begin with a survey of recent advances in the area of search and detection of smart and evasive targets by teams of searching agents. Next, we address topics focusing on the role of reinforcement learning in multi-agent search tasks in unknown environments and in adversarial settings, on task allocation between team members in multi-agent search tasks, on multi-robot exploration, and on the usage of reconfigurable team formations for search and surveillance applications. We then follow with a discussion on the implementation of multi-agent search tasks where despite imperfect communication between team members allow the team to reconfigure itself in order to complete the task. Furthermore, cooperative sensing and robot motion coordination, multi-agent active perception and multi-agent active search are addressed as important open problems in multi-agent search solutions in real-world scenarios. Figure 1 presents a diagram showing a classification of future research directions in multi-agent search tasks, addressed in this paper.

We hope that results achieved in previous works along with the outlining of intriguing future research directions regarding cooperation design in multi-agent search tasks will lead to further research in these directions.

## 2 A view on current research trends in multi-agent search

### 2.1 Introduction

Multi-agent search tasks aim at developing strategies for detection of targets in an area of interest. The targets being searched for can be static or mobile and information regarding the area in which the search is performed, the number of targets and their capabilities assumed in various studies ranges from complete knowledge of the search protocols to problems with targets having no, or very limited knowledge about the search process. There are several ways to categorize the approaches to search, however one major distinction between proposed methods are probabilistic approaches that aim to develop algorithms that maximizes the probability of detection and guaranteed detection protocols that aim to devise strategies that guarantee success of the search mission. These types of problems were investigated for centuries, and in modern times are typically considered to be carried out by teams of autonomous unmanned mobile agents such as UAVs or Unmanned Underwater Vehicles (UUVs). Among the pioneering works on this subject are Koopman (1980), Alpern and Gal (2006), and Stone et al. (2016).

Ismail et al. (2018), present a recent survey on current challenges and future research directions concerning cooperative multi-agent robot systems. The authors focus on three ingredients that specify and distinguish between cooperative multi-robot systems which are the types of agents, the control architecture and the communications method. Agents can either be identical (homogenous) or heterogeneous. Control architectures may be decentralized, centralized or a hybrid combination of the two. The communication method can be implicit or explicit.

Homogeneous agents allow easier and more efficient coordination, for some tasks using robots with different capabilities with having an ability to apply different algorithms may be more suitable. Communication between robots is a form of interaction that allows them to cooperate by, sharing and exchanging information and coordinate their actions to achieve a common goal. Communication can be done by the robot leaving pheromone traces in the environment and sensing the traces left by peers, interaction by explicit signaling by broadcasting messages to one another or implictly through the agents’ sensors, by reacting to the presence of other agents detected by a robot’s sensors.

The article surveys the possibilities of using centralized and distributed approaches to multi-agent systems design mentioning the tradeoffs between using global information to plan behaviors in a centralized way compared to having only local information, in distributed scenarios, which may impact the effectiveness of achieving the team’s goal. However, as the number of agents increases and the environment is changing in time, distributed approaches may be better suited since they require less communication computations and are faster, flexible and more adept to respond to changing situations.

In Chung et al. (2011b), a survey on search and pursuit-evasion in mobile robotics is provided. The review describes algorithms and results arising from different assumptions on searchers, evaders and environments and discusses potential field applications for the presented methods. Several pursuit-evasion games directly connected to robotics are the focus of the review, in contrast to the other cited surveys that concentrate on differential games. The paper concentrates on adversarial pursuit-evasion games on graphs and in polygonal environments where the objective is to maximize the worst-case performance on the search or capture time and on probabilistic search scenarios where the objective is the optimization of the expected value of the search objective, such as the maximal probability of detection or minimal capture time.

In Cao et al. (2012), an overview on distributed multi-agent coordination is provided. Among the reviewed topics that are addressed are consensus, formation control, optimization, and estimation. In Yan et al. (2013), a survey and analysis of multi-robot coordination is presented. The review categorizes papers based on their communication mechanism, planning strategies and their decision-making structure. In Chen and Barnes (2014), a review on human factors issues in human-agent teaming for multirobot control is provided. Aspects such as efficient human supervision of multiple robots, human trust in automated systems, maintenance of human operator’s situational awareness, human-agent interaction and retention of human decision authority are investigated. In Oh et al. (2015), a survey of recent results in multi-agent formation control is conducted. Existing works are classified according to position, displacement, and distance-based control.

In Khamis et al. (2015), a review on multi-robot task allocation is given. The paper describes recent approaches and future direction on how to optimally assign tasks to the members of a team of robots in order to optimize the team’s performance while satisfying to the constraints of the problem. The paper describes methods on how to model the problem such as discrete fair division, optimal assignment problem, alliance efficiency problem and the multiple travelling salesman problem. It further discusses categorization of task allocation schemes into single versus multi-task which describes the ability of robots to perform tasks in sequentially or in parallel. Single-robot versus multi-robot and instantaneous assignment versus time extended assignment which describes the planning performed by robots to allocate the tasks are additional categorization criteria.

In Robin and Lacroix (2016), a taxonomy and survey on multi-robot target detection and tracking is provided. Among the investigated topics are coverage, surveillance, search, patrolling, pursuit-evasion games, target detection and target tracking. Current approaches are described, and future open problems are discussed. In Cortés and Egerstedt (2017), a review on coordinated control of multi-robot systems is provided. Decentralized control and coordination strategies are investigated specifically in the context of developing decentralized algorithms that lead robot teams to be in specific geometric configurations by means of descent-based algorithms defined with respect to the performance of team. In Rizk et al. (2019), the current state of research in cooperative heterogeneous multi-robot systems is investigated. The review focuses on aspects such as task decomposition, coalition formation, task allocation, perception, and multi-agent planning and control. In Verma and Ranga (2021), a recent survey on multi-robot coordination analysis is provided.

In Drew (2021), a thorough survey on the current status of multi-agent systems for search and rescue applications is presented. The authors highlight the advancements made through the combination of machine learning and control techniques in perception driven autonomy, decentralized multi-robot coordination and human-robot interaction as the main fronts driving research forward. The paper further mentions that advancements in *ad hoc* networks are approaching the technological readiness level to enable reliable communication in safety-critical applications such as search and rescue missions. The survey further suggests that future research is essential in the context of simulation-to-reality (sim-to-real) transfer, for the purpose of enabling accurate models trained in simulation to be applicable in real world search and rescue scenarios as well. Furthermore, the authors indicate that verification and real-world evaluations of algorithms, especially ones that rely on machine-learning based technologies and ones that have some degrees of autonomy should be more extensively tested in the field to allow a true understanding of the performance and maturity of these potentially life-saving technologies.

In Queralta et al. (2020), collaborative multi-robot planning, coordination, perception and active vision are investigated in the context of search and rescue applications. The paper provides a review on heterogeneous search and rescue robots in different environments and on active perception in multi-robot systems. The paper discusses the most pressing open problems in these topics which are shared autonomy, the ability to transfer knowledge gained in simulation to reality (sim-2-real), coordination and interoperability in heterogeneous multi-robot systems, and active perception in a variety of environments such as maritime, urban, wilderness or disaster zones.

The paper interestingly describes challenges and opportunities for autonomous robots in different types of environments such as urban, maritime and wilderness search and rescue missions and discusses the types of heterogeneous autonomous robots that are used in different search and rescue settings as well as the advantages of each robot and abilities of each type of robot to complement the capabilities of different types of robots throughout the mission.

An additional aspect that the paper discusses is shared autonomy and human-swarm interaction. Shared autonomy refers to the autonomous control of the majority of degrees of freedom in a system, while designing a control interface for human operators to control a reduced number of parameters defining the global behavior of the system. Furthermore the paper discusses the important role of communication by using robotic mobile *ad hoc* networks for the purpose of coordination and sharing of information between team members, highlighting that maintaining communication connectivity between the robots is essential in search and rescue missions, a topic that has been extensively studied in decentralized multi-agent control applications such as Barel et al. (2019) and Segall and Bruckstein (2022).

The survey continues to describe the main algorithm types that are essential to multi-robot coordination and planning for collaborative robotic search and rescue missions. Amongst them are multi-robot task allocation, path planning and area coverage, area exploration and centralized and distributed multi-robot planning.

In Williams (2020), a different perspective than the ones discussed in this paper concerning collaborative multi-robot multi-human teams in search and rescue applications is investigated. The paper addresses several goals in this topic which are: a path planner for multiple UAVs that is adaptable to human behaviour, behavioural modeling that allows to localize locations of lost people, a distributed computing prototype that allows UAVs to perform calculations and communications, as well as an interface allowing human-robot interaction for various levels of robot autonomous capabilities. Enabling autonomous robots to perform search and rescue efforts reduces the cost of these search missions, allows area to be searched faster and allows robots to explore areas that are inaccessible to humans. The authors pose interesting questions regarding the exchange of information between robotic searchers and the human controller and debate on the balance between remotely controlling the UAV team and letting them to autonomously collaborate and explore the area. Therefore, the solution that is sought seeks to achieve maximal search effectiveness with minimal cost and computational capabilities by developing a time-varying policy that allows humans and robots to work together in order to perform efficient decision making by utilizing both human and robot resources alike.

In Li et al. (2020), A path planning method for sweep coverage with multiple UAVs that act as a wireless sensor network is investigated, discussing an additional future research domain related to multi-agent surveillance algorithms. The paper considers the sweep coverage problem, in which several UAVs serve as mobile sensors and are used to patrol an area in search of targets. The goal is to allow maximal coverage of targets with the fewest number of UAVs. Since the battery lifetime of UAVs is constrained, they do not have the required energy to allow them to visit all targets before they have to land to recharge. Therefore, targets are ranked according to their importance and targets are chosen to be visited accordingly. The coverage rate is defined as the ratio between the total weights of covered targets and the total weights of all targets. Since maximizing target coverage and minimizing search time are opposing objectives, a new weighted targets sweep coverage algorithm is proposed allowing the construction of effective flight paths for UAVs to solve the min-time max-coverage problem.

In Rahman et al. (2021), run-time monitoring of machine learning algorithms for robotic perception is investigated. Run-time monitoring determines the performance of a robot in test scenarios and in environments and conditions it did not previously encounter in training in order to test its true behaviour in the field and its generalization capabilities. This is an extremely important topic regarding the applicability of using on-board machine learning algorithms in real-world tasks, specifically aboard drones. Machine learning (ML) tasks such as perception tasks are often very energy consuming (Deng, 2018; García-Martín et al., 2018; García-Martín et al., 2019), thus they deplete the battery of drones faster, and limit the time they can be airborne. This occurs since ML algorithms require heavy computations, resulting in the need to carry larger batteries with increased energy capacity that in turn lead to an increased weight of a robot’s payload, to meet its power demand.

In Castrillo et al. (2022) a review on anti-unmanned aerial systems technologies for cooperative defensive teams of drones is provided. The paper discusses the concept of a multi-robot team that acts as a cooperative defensive system against other drones. To facilitate the development of such anti-UAV methods, the recent technological status for sensing, mitigation and command and control systems for intercepting unmanned aerial systems are discussed, as well as their applicability for usage in mini drones. In Barbeau et al. (2022) recent trends on collaborative drones are explored with an emphasis on surveying threats posed by drones, target recognition, navigation under uncertainty and risk avoidance which are relevant topics in the perspective of multi-agent search tasks.

In Horyna et al. (2022), decentralized swarms of unmanned aerial vehicles for real-world search and rescue operations without explicit communication are explored. A self-adaptive communication strategy is developed to allow an efficient change of swarm azimuth to a direction with a higher priority in order to inspect an object of interest based on the real-time on-board detections. A local visual communication channel allows neighboring robots without explicit communication to achieve high reliability and scalability of the system. The developed techniques can aid in victim verification and close-range inspections and can also be applied for cooperative environment exploration.

At last we discuss the topic of learning sub-team performance as an additional future research direction for advancing multi-robot searching systems. In Banfi et al. (2022), hierarchical planning for heterogeneous multi-robot routing problems *via* learned sub-team performance is examined in a different context than search and rescue applications however ideas drawn from the paper may be applied in this context as well.

### 2.2 Probabilistic search and detection of opponents

This section highlights works concerned with probabilistic search and detection of opponents. This family of approaches aims to develop algorithms that maximize the probability to detect a set of targets being searched. These types of problems are often referred to as pursuit-evasion games, in which the pursuers’ goal is to detect and catch the evaders and the evaders goal is to avoid being detected and caught by the pursuing team. There are several variants of pursuit-evasion games which include different combinations of single and multiple evaders and pursuers settings.

In Chung et al. (2011a), optimal detection of an underwater intruder in a channel using unmanned underwater vehicles is considered. The objective is to devise a set of periodic trajectories that are implemented by the patrolling unmanned underwater vehicles that aim to maximize the probability to detect the intruder. The problem is solved by using tools from optimal control theory. In Gan and Sukkarieh (2011), multi-UAV probabilistic target search using explicit decentralized gradient-based negotiation is explored.

Developing strategies for multi-agent perimeter patrol in adversarial settings where an opponent possesses full knowledge of the patrolling agents’ search strategy are developed in Agmon et al. (2008) and Agmon et al. (2011). Possessing information about the agents’ patrol strategy allows the smart opponent to attempt entering the guarded perimeter at the location with the highest probability for its success at being undetected. To prevent the opponent from utilizing its knowledge on the patrol strategy, randomness is introduced into the robots’ perimeter patrol algorithm, hence preventing the opponent from having full knowledge of the chosen patrol strategy and thus increasing the chances of the patrolling team to detect it.

There is also a large body of literature concerning search for targets in discrete domains. In Casbeer et al. (2013) a UAV is charged with the task of estimating the state of an intruder by gathering information from unattended ground sensors that relay information when the UAV is in close proximity to them. The transmitted information includes describing whether an intruder passed in the vicinity of the sensor, the time at which the intruder was detected as well as its speed. The intruder’s position and speed are estimated and the network on which the intruder moves is a discrete occupancy grid. In Ahmed et al. (2017), an additional work that considers probabilistic multi-target localization on road networks by using unattended ground sensors is investigated. In Temple and Frazzoli (2010) a somewhat related problem to discrete search for smart targets, the cow path problem is investigated. The cow path problem, is an on-line search problem where *k* short-sighted cows search for a reward on *m* (*k* ≤ *m*) paths which diverge from a single origin and do not intersect each other. The cows goal is to get the reward at the minimal grazing time. The solution is provided by minimization of the expected time until a cow reaches the reward.

In Makkapati and Tsiotras (2019), pursuit–evasion problems involving multiple pursuers and multiple evaders (MPME) are investigated. Pursuers and evaders are all assumed to be identical, and pursuers employ either, constant bearing or pure-pursuit strategies. The problem is simplified by using a dynamic divide and conquer approach, where at every time instant each evader is assigned to a set of pursuers based on the instantaneous positions of all the players. The original MPME problem is decomposed to a sequence of simpler multiple pursuers single evader (MPSE) problems by checking if a pursuer is relevant or redundant against each evader. Then, only relevant pursuers participate in the MPSE pursuit of each evader. Recent surveys on pursuit evasion problems are Chung T. H. et al. (2011), Kumkov et al. (2017), and Weintraub et al. (2020).

In Kumkov et al. (2017), a survey on pursuit problems with 1 pursuer versus 2 evaders or 2 pursuers versus 1 evader are formulated as a dynamic game and solved with general methods of zero-sum differential games. In Weintraub et al. (2020), a survey on pursuit-evasion differential games that classifies papers according to the numbers of participating players: single-pursuer single-evader (SPSE), MPSE, one- pursuer multiple-evaders (SPME) and MPME is provided.

In Garcia et al. (2019), a two-player differential game where a pursuer attempts to capture an evader before it escapes a circular region is explored. The state space, comprised of pursuer and evader locations, is divided into evader and defender winning regions. In each region the players try to execute their optimal strategies. The players’ strategy depends on the state of the system (if it is in the capture or escape regions), and the proposed approach guarantees that if the players execute the prescribed optimal moves they improve their chances to win. The players move at a constant speed and the pursuer is faster than the evader. The players’ controls are the instantaneous heading angles. The game is a two-termination set differential game, i.e., the game ends when either player wins. In Garcia et al. (2020), the problem of a border defense differential game where *M* pursuers cooperate to optimally catch *N* evaders before they reach the border of the region and escape is investigated. The pursuer team exchanges information between its team members in order to determine the discrete assignment of pursers to evaders in an on-line manner. Furthermore, the game is a perfect information differential game where both pursuers and evaders have access to all state variables, which are the locations of all players, as well all their dynamics and velocities. The pursuers in this setting are assumed to have greater speeds than the evaders. The game takes place in a simple half-plane environment, and ends when all evaders are either caught or reach the border and escape.

Pursuit-evasion games are applied to address defending a region from the entrance of intruders as well. Such works are Shishika and Kumar (2018), Shishika et al. (2019), and Shishika et al. (2020) which investigate perimeter defense games and focus on cooperation between pursuers to improve the defense tactic.

In Khaluf et al. (2018), the authors investigate exploration of unknown environments by an autonomous team of robots that perform a collective Lévy walk to efficiently explore the area. The motivation to perform Lévy walks for exploration tasks in environments where no prior knowledge exists on the location of the targets being searched is that it maximizes the search efficiency, implying that for a given time interval the number of detected targets is maximal compared to other types of random walks and exploration strategies such as Brownian motion. In a Lévy walk step the orientation is sampled from a uniform distribution and the length of the step from a heavy-tailed distribution.

The main contribution of this paper is to preserve the Lévy properties of the walk for a large swarm of searching robots, which tend to disappear as the number of robots in the swarm increases due to the intersecting paths of the robots. The developed algorithm strives to perform a Collective Lévy Walk for the entire swarm by considering local communication between searching robots, thus improving the efficiency of the search compared to an algorithm in which each robot performs an explorative Lévy walk independently without coordinating its actions with the other members of the searching swarm. This is achieved by using an artificial potential field that repels spatially close robots from each other in order to increase the length of the walk they perform. Increasing the length of the walks enables the robots to explore more areas in less time, and prevents them from visiting targets it already found, thus resulting in more detected targets.

The results of the experiments conducted in the paper, indeed show that the resulting distribution of robot walk lengths for large robot swarms implementing the Collective Lévy Walk is closer to a heavy-tailed distribution compared to an exponential distribution. This is in contrast to swarms that execute independent Lévy walks in which the distribution of walk lengths does not follow a heavy-tailed distribution for increasing numbers of searching robots.

In Nauta et al. (2020), the authors investigate a similar problem of searching for a set of targets in an unknown environment by utilizing a team of searching robots. The robots perform a collective Lévy walk that preserves the desired properties of the Lévy walk for large swarm sizes of several hundreds of searching robots. The authors investigate search of targets that are homogeneously dispersed in the explored environments as well as scenarios in which the targets are dispersed heterogeneously and are located in separated robot clusters across the environment.

In addition to maximizing the efficiency of the search, another metric that is calculated to determine the quality of the search is the fraction of targets detected compared to the total number of targets that are present in the environment. For heterogeneous environments patch search efficiency is also explored in order to measure the quality of detections within each target patch. The computed metrics aim to capture both the diffusiveness of the searching swarm in the area and its collective efficiency in detecting targets.

The investigated protocols are the individual Lévy walk, the Collective Lévy walk and the Adaptive Lévy walk that extends the collective Lévy walk algorithm to heterogeneous environments and increases its efficiency. The Adaptive Lévy walk achieves this performance increase by adapting the Lévy parameter *α* that controls the length of the walk step, based on a local density estimation performed by each robot. Once a target is detected, the robot infers that it is likely next to a cluster of robots and hence assumes the density of targets in its vicinity is high.

Therefore, by changing *α* and setting *α* = 2, which implies the robot is performing Brownian motion, improves the detection capability since Browning motion is known to be optimal in environments with high target density, as proved in Bartumeus et al. (2002). Therefore, by adapting its walk length based on the sensed target density distribution the Adaptive Collective Lévy walk achieves better results compared to the compared protocols while preserving the desired properties of the Lévy walk for swarms with a large number of searching robots.

### 2.3 Guaranteed detection of smart opponents

A smart target is an opponent that can perform optimal evasive maneuvers meant to avoid its interception. The works discussed in this section aim to provide an extensive and complete theoretical, analytical and experimental framework for search tasks involving unknown numbers of smart targets. Planning against an unknown number of opponents relates to problems of planning under uncertainty and planning without complete state information and is a scenario that is seldom investigated in the context of adversarial search tasks as most works assume some prior knowledge on the number of opponents and their locations.

The searching agents which are referred to as sweepers are equipped with sensors whose purpose is to detect the targets/opponents. Throughout this section the terms searchers and sweepers are the same and both refer to the searching team of agents that attempts to catch all evaders. In recent years, swarming technologies are attracting attention as a means of successfully accomplishing complex goals by relying on redundancy and robustness to failures by employing a large number of simple robots with limited capabilities instead of using a small number of sophisticated and expensive robots. The design and implemented protocols of the robot teams discussed in this part draw inspiration from such robots.

In Vincent and Rubin (2004), investigate guaranteed detection of smart targets in a channel environment using a team of detecting sweeping agents and Altshuler et al. (2008) provides optimal strategies to the same problem. In McGee and Hedrick (2006), investigate search patterns for the purpose of detecting all smart evaders in a given planar circular region. The intruders do not have any maneuverability restrictions besides an upper limit on their speed. The searchers are equipped with sensors that detect evaders that are inside a disk shaped region around them. The considered search pattern is composed of spiral and linear sections.

In Tang and Ozguner (2006), propose a non-escape search procedure for evaders. Evaders are originally located in a convex region of the plane and may move out of it. Tang et al. propose a cooperative progressing spiral-in algorithm performed by several agents with disk shaped sensors in a leader-follower formation. The authors establish a sufficient condition for the number of searching agents required to guarantee that no evader can escape the region undetected.

In Guerrero-Bonilla et al. (2021a), the authors investigate area defense and surveillance on rectangular regions using control barrier functions to develop a guaranteed defense strategy in which a defending robot ensures that an intruder cannot enter the defended region by applying appropriate reactive control laws. In Guerrero-Bonilla et al. (2021b), a control law based on control barrier functions is applied for robust perimeter defense. In this work a team of robots move around the perimeter of the region in order to guarantee detection of an intruder that attempts to enter the defended region. In Guerrero-Bonilla and Dimarogonas (2020), a solution to the perimeter surveillance problem for one intruder and several defending robots based in set-invariance is provided.

**FIGURE 2:**
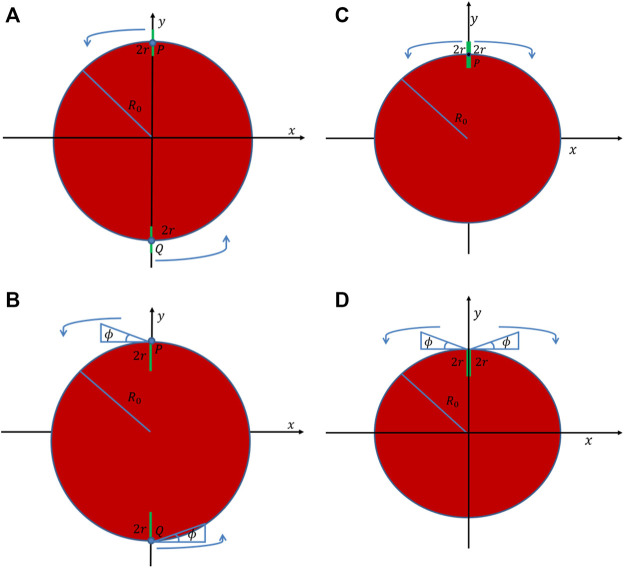
**(A)**—Initial placement of two agents employing the same-direction circular sweep process. **(B)**—Initial placement of 2 agents employing the same-direction spiral sweep process. **(C)**—Initial placement of 2 agents employing the circular pincer sweep process. **(D)**—Initial placement of 2 agents employing the spiral pincer sweep process. The sweepers sensors are shown in green. The angle *ϕ* is the angle between the tip of a sweeper’s sensor and the normal of the evader region. *ϕ* is an angle that depends on the ratio between the sweeper and evader velocities.

First, several search challenges for detection of smart evaders are presented. In all these problems the aim is to develop a “must-win” strategy that does not allow any evaders to escape the scanned area without being detected by the sweepers. The team of sweepers attempts to achieve this goal by sweeping around and inside a region in which the smart evaders are known to be initially located. In the considered problems this region is chosen to be a circular region of radius *R*_0_ as depicted in Figure 2, that does not have any physical boundaries, implying that evaders may attempt to move out of the region in order to escape the team of searchers. Hence, as the search protocol progresses, the region that might contain potential evaders increases.

There can be two goals for the sweeping agents. The first is the confinement task. The confinement task is a task in which sweepers ensure that the “evader region” (the region where evaders may possibly be located) does not increase by sweeping around the region. The second task is the cleaning or detection/coverage task. In this task, after each sweep, the sweepers move into the evader region while also sweeping around it, thus ensuring the eventual detection of all evaders initially residing in the region. We consider teams of agents that sweep several different search patterns. In our recent works, we analyzed several different search strategies based on circular sweeps, spiral sweeps and most importantly pincer-based sweeps that thoroughly investigate the topic of guaranteed detection of smart opponents by multi-robot teams. The sections below discuss the main results and insights that were gained in our latest research.

Evaders attempt to escape the searching team and move out of the initial region, at a maximal speed of *V*_*T*_. All sweepers move at a speed *V*_*s*_ > *V*_*T*_ and detect evaders with linear sensors of length 2*r*. A linear sensor of length 2*r* is a rectangular shaped sensor with practically zero width and a length of 2*r* and may be considered as a one-dimensional linear sensor array with a length of 2*r*. This array structure is highly common in many sensing and scanning applications, from optical to radar and sonar. We chose to analyze the performance of the system when the sensor is a linear array as this type of sensor is highly common as indicated above and moreover it presents the simplest case of a standard rectangular sensor found in any camera.

Every evader that intersects a sweeper’s field of view is immediately detected. There can be any number of evaders inside the region, and this number as well as the evaders’ locations is unknown to the sweepers. Every “must-win” strategy requires a minimal speed that depends on the trajectory of the sweepers. We evaluate the different strategies by using two metrics, total search time until all evaders are detected, and the minimal critical speed required for a successful search.

The critical speeds developed for each type of sweeping strategy are compared to a lower bound on the sweepers’ speed that is independent of the particular choice of search method and depends only on the geometric properties of the region, the evaders’ speed, the number of sweepers in the search team and the geometry of the sweepers’ sensors. We use this lower bound to compare between the different search methods and evaluate how close they are to an optimal solution. This lower bound is derived in Francos and Bruckstein (2021a) and the considerations that lead to the established results are outlined in the next paragraph. For a full proof see Section 3 of Francos and Bruckstein (2021a).

The maximal cleaning rate occurs when the footprint of the sensor over the evader region is maximal. For a line shaped sensor of length 2*r* this happens when the entire length of the sensor fully overlaps the evader region and it moves perpendicular to its orientation. The rate of sweeping when this happens has to be higher than the minimal expansion rate of the evader region (given its total area) otherwise no sweeping protocol can ensure detection of all evaders. We analyze the search process when the sweeping team consists of *n* identical agents. The lowest sweeper speed satisfying this requirement is defined as the critical speed and is denoted by *V*_*LB*_, Hence:

Theorem 1 No *sweeping protocol may successfully accomplish the confinement task if its speed*, *V*_*s*_, *is less than*,

$$V_{LB}=\frac{\pi R_{0}V_{T}}{nr}$$ (1)

The complete search time until all evaders are detected depends on the search protocol performed by the team of sweepers. Two types of search patterns are investigated, circular and spiral, for any even number of sweepers. The desired outcome is that after each sweep around the region, the radius of the circle bounding the evader region (for a circular sweep) or the actual radius of the evader region (for a spiral sweep) is reduced by a strictly positive value. This guarantees complete detection of all evaders, by decreasing in finite time the potential area where evaders may be located to zero. At the start of the circular search protocol only half the footprint of the sweepers’ sensors is inside the evader region, i.e., a footprint of length *r*, while the other half is outside the region with the intention of detecting evaders that attempt to escape outside of the region. At the start of the spiral search protocol the entire length of the sweepers’ sensors is inside the evader region, i.e., a footprint of length 2*r*.

In Francos and Bruckstein (2021b), we proved that a smart evader may escape from point *P* = (0, *R*_0_) (shown in Figure 2A), when basing a single sweeper’s speed only on a single traversal around the evader region. Hence, we had to increase the sweeper’s critical speed to cope with such a potential adversarial escape plan. Point *P* is considered as the “most dangerous point,” meaning that it has the maximum time to spread during sweeper movement, hence if evaders spreading from this point are detected, evaders that attempt to escape from all other points will also be detected. If we choose to distribute a multi-agent search team equally along the boundary of the initial evader region, we would have the same problem of possible escape from the points adjacent to the starting locations of every sweeper.

In Francos and Bruckstein (2021a) we proposed an alternative method for multi-agent search strategies in which pairs of sweepers move out in opposite directions along the boundary of the evader region and sweep in pincer-movements instead of deploying sweepers at equally spaced intervals along the boundary and requiring them to move in the same-direction. The proposed search protocol can be employed by a search team with any even number of sweepers.

At the start of each sweep, sweepers are positioned in pairs back-to-back. In each pair, one sweeper moves counter-clockwise while the other moves clockwise. Every time sweepers meet, implying that their sensors are back-to-back again, they exchange their movements directions. The search region is partitioned into a number of non-overlapping sections that depend on the number of sweepers in the search team, such that every sweeper sweeps a particular angular sector of the evader region.

It is worth emphasizing that once a sweeper leaves a location that was cleared from evaders, other evaders may attempt to reach this location again. Therefore, considered sweep protocols must ensure that there is no evaders strategy that enables any evader to escape even if evaders wait at the edge of a cleared location and start their escape instantly after a sweeper leaves this location.

Sweeping with pincer-based search protocols removes the need to sweep additional areas to detect evaders from these additional “most dangerous points” since in pincer-based protocols the “most dangerous points” are now located at the tips of their sensors closest to the evader region’s center and not on the boundary of the evader region as occurs in same-direction sweep protocols.

The described search protocols can be either 2 dimensional where sweepers travel on a plane or 3 dimensional implying that sweepers are drone-like agents that fly over the evader region. In case the search is planar, exchanging of movement directions takes place after the completion of each sweep when a sweeper “bumps” into a sweeper that scans the adjacent section. If the search is 3 dimensional, sweepers fly at different altitudes above the evader region, and every time a sweeper is directly above another, they exchange the angular section they are responsible to sweep between them, and continue the search. The analysis of 2 and 3 dimensional search protocols is similar.

In Francos and Bruckstein (2021a) we presented an extensive theoretical evaluation of pincer sweep methods for detection of smart evaders and provided analytical results for sweep times and critical speeds of the different proposed search strategies. We list the main theorems and results to enable a more complete discussion on the established results.

Here, we shall address a quantitative and qualitative comparison analysis between the total search time of same-direction sweep processes and pincer-movement search strategies. We shall evaluate the different strategies by using two metrics, total search time and the minimal critical velocity required for a successful search. We compare two types of pincer-movement search processes, circular and spiral, with their same-direction counterparts, for any even number of sweeping agents.

Figure 2 shows the initial placements of sweepers employing circular and spiral search tasks for same-direction and pincer-based search strategies.

**FIGURE 3:**
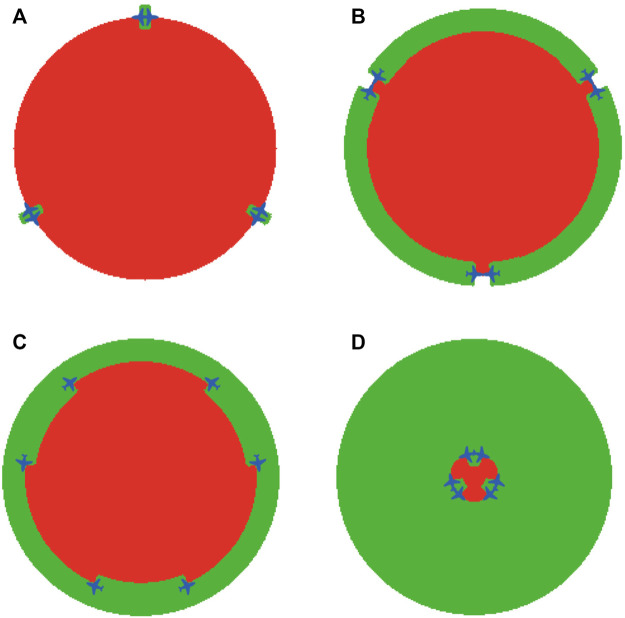
Swept areas and evader region status for different times in a scenario where 6 agents employ the circular pincer sweep process. **(A)**—Beginning of first cycle. **(B)**—Toward the completion of the first cycle. **(C)**—Beginning of the second cycle. **(D)**—Beginning of the one before last cycle. Green areas are locations that are free from evaders and red areas indicate locations where potential evaders may still be located.

Figure 3 presents the swept areas and evader region’s status that results from a dynamical NetLogo Tisue and Wilensky (2004) simulation of a circular pincer sweep process performed by 6 sweepers. Green areas are locations that were detected by the sweeping team and do not contain evaders at the current time instance. Red areas indicate location in which potential evaders can still be present.

**FIGURE 4:**
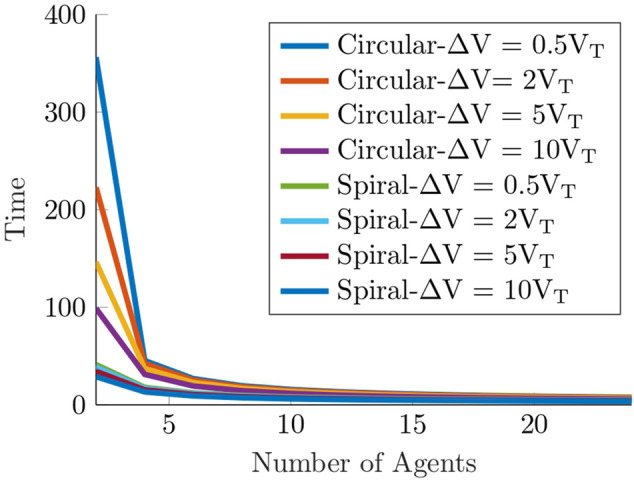
Total search times until complete cleaning of the evader region for the circular and spiral sweep processes. We simulated sweep processes with an even number of agents, ranging from 2 to 24 agents, that employ the multi-agent circular and spiral sweep processes. We show results obtained for different values of speeds above the circular critical speed of 2 sweepers. The chosen values of the parameters are *r* = 10, *V*_*T*_ = 1 and *R*_0_ = 100.

In order to make a fair comparison between the total sweep times of sweeper teams that can perform both the circular and spiral sweep processes, the number of sweepers and sweepers’ speed must be the same in each of the tested spiral and circular teams. As proven in Francos and Bruckstein (2021a), the critical speed required for sweepers performing the circular sweep process is higher than the minimal critical speed required for sweepers performing the spiral sweep process. Therefore, in Figure 4 we show the spiral sweep process’s sweep times that are obtained for different values of speeds above the circular critical speed. This means that the values of Δ*V* that are mentioned in the plots correspond to sweeper speeds that are almost twice the spiral critical speeds.

Figure 4 compares the cleaning times of circular sweeping teams and spiral sweeping teams consisting of 2–24 sweepers. The results are computed with the same sweepers speed for both the circular and spiral multi-agent sweep protocols. The reduction in complete sweeping times that are achieved when sweepers employ the spiral search process are clearly observable. This result is independent of the number of the sweepers that perform the search or the speed in which they move.

**FIGURE 5:**
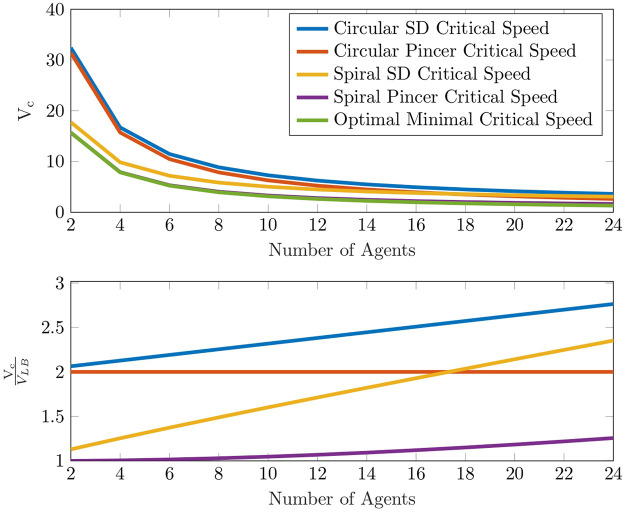
Critical speeds as a function of sweepers’ numbers.

Figure 5 provides a comparison between critical speeds of same-direction and pincer-based search protocols for guaranteed detection of smart evaders. Results show the superiority of using pincer-based approaches since they result in lower critical speeds compared to their same-direction counterparts. Furthermore, the results show that for an increasing number of sweepers, circular pincer-based protocols require a smaller critical speed even when compared to spiral same-direction protocols that can only be implemented with sweepers that have more advanced capabilities that allow them to accurately track the expanding wavefront of the evader region instead of performing a simple circular pincer-based movements. The top plot shows the critical speeds of each protocol as a function of the number of participating sweepers. The bottom plot presents the ratio between the critical speeds of the different guaranteed detection investigated protocols and lower bound on the critical speed presented in Theorem 1. It can be observed from the figure that the ratio between the spiral pincer critical speed and the lower bound is almost 1 emphasizing that it is nearly optimal.

In Francos and Bruckstein (2022a), we look at a somewhat dual problem to the previously considered problem. This work examines the problem of defending a region from the entrance of smart intruders or invaders. Sweepers may perform outwards expansion from the initial “safe” area until the sweepers reach the maximal radius of a circular area that they can protect given their predetermined speed. This extension investigates both multi-agent circular and spiral defense protocols.

It focuses on developing a guaranteed defense protocol of an initial region from the entrance of an unknown number of smart invaders. The region is protected by employing a multi-agent team of identical cooperating defenders that sweep around the protected region and detect invaders that attempt to enter it. The defenders possess a linear sensor of length 2*r*, similar to the one described in the previous section, with which they detect invaders that intersect their field-of-view. The only information the defenders have is that invaders may be located at any point outside of an initial circular region of radius *R*_0_, referred to as the initial protected region at the beginning of the defense protocol.

There are two objectives for a defense strategy, defending the initial protected region and, if possible, expanding the protected region by performing an iterative expansion strategy until the region reaches the maximal defendable area.

Successfully completing the defense and maximal expansion tasks with the lowest possible critical speed is one of the performance metrics used as a benchmark for having an efficient defense strategy. Pincer-based defense procedures result in lower critical speeds compared to their same-direction counterparts, and hence are chosen in the developed defense protocols. The discussed pincer-based strategies can be performed with any even number of defenders. Based on the numbers of defenders performing the defense task, the protected region is portioned into equal angular sectors, where each sector is searched by a different defender.

**FIGURE 6:**
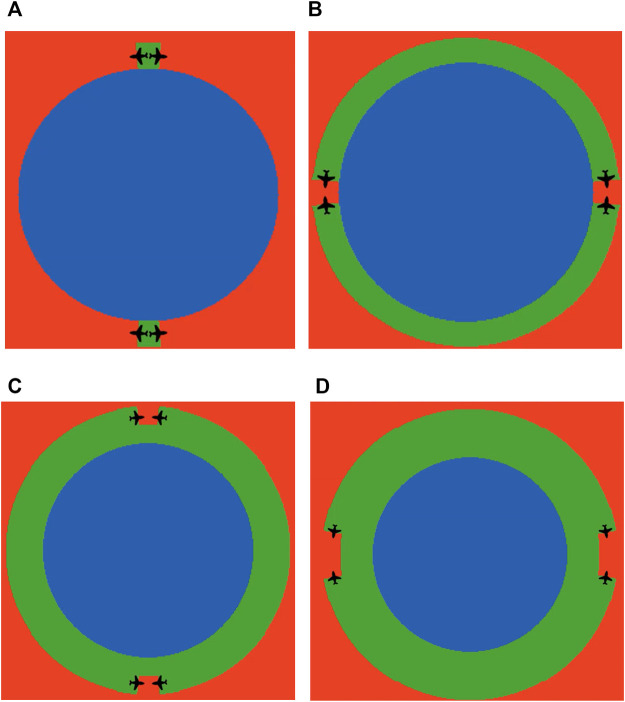
Swept areas and protected region status for different times in a scenario where 4 defenders perform the spiral defense pincer sweep protocol. **(A)**—Beginning of first sweep. **(B)**—End of the first sweep. **(C)**—End of the second sweep. **(D)**—Toward the end of the third sweep. Green areas show locations that were searched and hence do not contain invaders and red areas indicate locations where potential invaders may be present. Blue areas represent locations that belong to the initial protected region that does not contain invaders.

Figure 6 shows the evolution of the defense protocol during the expansion of the protected region carried out by 4 defenders implementing the spiral defense pincer sweep protocol. Green areas indicate locations that defenders already cleared from invaders. Hence, these areas do not contain invaders at the current time instance. Green areas may become red again due to the advancement of invaders from the exterior region, once defenders continue to sweep different areas and their sensors detect invaders elsewhere. Red areas indicate locations where potential invaders may be present while blue areas represent locations that belong to the initial protected region that does not contain invaders.

Note that in the considered problems, the search is continued until the expansion of the protected region reaches the maximal attainable radius, and afterwards the defenders continuously patrol around this radius.

**FIGURE 7:**
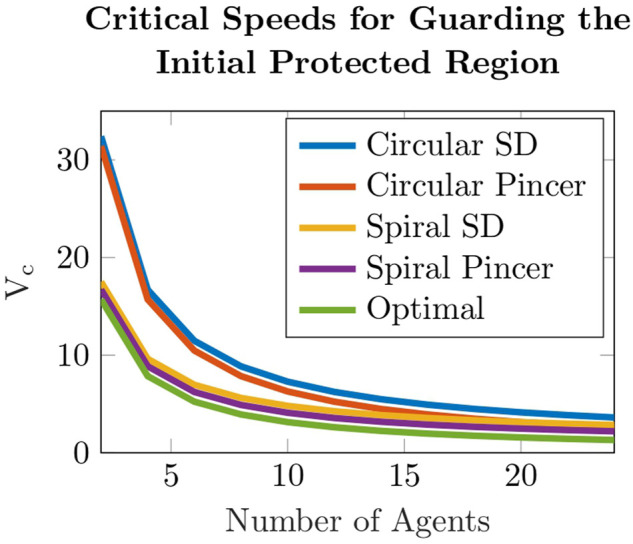
Critical speeds as a function of the number of defenders. The number of defenders is even, and ranges from 2 to 24 defenders, that perform the spiral and circular defense pincer sweep protocols as well as the spiral and circular defense same-direction sweep protocols. The optimal lower bound on the critical speeds is presented for comparison as well. The chosen values of the parameters are *r* = 10, *V*_*T*_ = 1 and *R*_0_ = 100.

Figure 7 presents critical speeds as a function of the number of defenders. The number of defenders is even, and ranges from 2 to 24 defenders, that perform the spiral and circular defense pincer sweep protocols as well as the spiral and circular defense same-direction sweep protocols. The optimal lower bound on the critical speeds is presented for comparison as well. Development of better defense strategies enables the defending team to expand the protected region to a larger area and to achieve this expansion in minimal time.

In Francos and Bruckstein (2022b), the guaranteed detection problem is formulated as a resource allocation problem from the perspective of the designer of the sweeping team.

When designing a robotic system composed of one or more robots the designer must consider the most cost-effective solution to the problem. In this context we choose to focus on one such aspect in the design, namely, the sensing capability, or the visibility range of the searchers. This criterion translates into solving the surveillance problem with a large number of simple and relatively low-cost agents equipped with basic sensing capabilities or alternatively, with a small number of sophisticated and expensive agents equipped with more advanced and accurate sensors. Taking this approach to the extreme can be seen as choosing to survey a region with a satellite equipped with a high-resolution camera or surveying the same region with multiple UAVs that fly at lower altitudes, carrying lower resolution cameras, in order to achieve effectively the same spatial coverage.

Such considerations are present across multiple domains of surveillance and monitoring applications such as security, search and rescue, crop monitoring, wildlife tracking, fire control and many more. When searching an area for evaders, this manifests in choosing to scan the area with fewer agents equipped with higher resolution sensors, compared to scanning the area with more agents having lower sensing capabilities. The mentioned research addresses such questions both from a theoretical and a practical perspective.

### 2.4 Reconfigurable team formations for search and surveillance applications

Multi-Agent formation control refers to the ability to control and coordinate a multi-robot team to preserve a specific structure or configuration throughout its movement. Moving in a specific structure that allows to preserve relative distances and orientation between agents or allowing a multi-robot team to reconfigure itself and change its configuration into a more suitable one for the task it wishes to accomplish is a very useful property for multi-agent search applications. Possible use cases are the ability of the team to pass through narrow corridors, which is essential in underground search operations or in scenarios where a specific distance must be maintained between agents to avoid losing connectivity between the team’s members.

The main challenges that appear when trying to control the formation of a multi-robot team are its stability, controllability of different formations, reconfiguration of formations, assignment of roles inside the formation as well as safety and uncertainty concerns Ismail et al. (2018). Formation control algorithms can be considered as part of multi-agent path planning algorithms that adhere to a specific structure the team’s member follow while they move towards their goal. Formation control algorithms are generally divided based in the control variables that are measured and used to steer and control the formation. These are position-based, displacement-based control and distance or bearing only based control.

According to the survey in Ismail et al. (2018) there are three main strategies for formation control used in multi-agent robotic settings. These are behavior-based, virtual structure and leader follower settings. Behavior-based approach refers to several desired behaviors of the agents such as goal seeking, obstacles avoidance, collision avoidance, etc. The advantage of this approach is it can be used to guide the multi-agent robots in the unknown or dynamic environment by using only local information available to robots. The drawback of using such solutions is that there are no theoretical guarantees or optimality of the solution since it relies on average behavior of the agents.

Virtual structure refers to a formation control paradigm that views the entire formation as a rigid body. This allows easier control of the formation at the expense of its flexibility. Leader- follower formation control allows leaders to advance toward their goal while the objective of the followers is to keep the structure of the formation by orienting themselves accordingly throughout the movement. The drawback for using this approach is that the entire formation depends on the movements of the leader and if it malfunctions then so does the rest of the formation.

Formation control algorithms designed for outdoor environments usually rely on the precise global positions of agents often use a centralized control architecture and collaborative decision making that leverages the availability of such rich information. This however, comes at the expanse of less flexibility and with a greater computational burden. Distributed and decentralized approaches that control the displacement between agents, distance or bearing-based control, on the other hand, enable more distributed implementations with only local interactions among the different agents.

Formation control algorithms are used in search and rescue missions to enable usage of multi-robot *ad hoc* networks, see Lin et al. (2014) and to multi-robot surveillance and monitoring tasks such as the approach presented in Borkar et al. (2020), where reconfigurable formations of quadrotors on Lissajous curves for surveillance applications are investigated. In an earlier work Borkar et al. (2016), the authors proposed using a multi-agent formation with constant parametric speed to achieve repeated collision-free surveillance and guaranteed area coverage in order to detect and entrap targets. In their recent work Borkar et al. (2020), local cooperation between agents allows to achieve collision free reconfiguration between different Lissajous curves allowing to handle scenarios such as agent addition, agents removal and agent replacement which are essential in surveillance applications.

In Xu et al. (2022), event-triggered surrounding formation control of multi-agent systems for multiple dynamic targets is investigated from a control perspective. Surrounding formation control scenarios aims to provide an algorithm in which agents encircle single or multiple targets that can either be idle or dynamic. The proposed control algorithm aims to achieve diverse surrounding patterns with both monolayer and multilayer configurations which have distinct benefits to various application scenarios such as escorting, patrolling, search and rescue and surveillance. Since previous surrounding control protocols rely on continuous information exchange between agents, they do not scale well to increasing number of agents and become more challenging due to communication burden in dynamic and unknown environments where continuous information needs to be shared between the formation’s agents. Hence using event-triggered control paradigms allows to reduce the communication overload between agents and to only periodically send information that allows the agents to update positions.

### 2.5 Reinforcement learning in multi-agent search tasks

Reinforcement learning is a branch of machine learning that is concerned with enabling a set of agents to learn to take actions by interacting with a dynamic environment. The agents’ goal is to maximize a cumulative reward that is received based on the actions the agents perform. The feedback to the agent’s actions is obtained through the reward signal, and the exploitation concept is applied to choose the next action. Agents can also choose to explore the environment in order to discover additional or better action candidates. This is known as the exploration concept. Many reinforcement learning approaches use a dynamic programming technique. Hence, one of the main objectives in developing an efficient policy is the ability of agents to estimate the importance of transitioning to a specific state.

A reinforcement learning model consists of 5 elements. The set of states, *S*, the set of actions *A*, the policy or mechanism that allows transition between the states, *P*: *A* × *S* → [0, 1], usually given by the probability of taking an action *a* while at state *s*; a scalar reward of that is received by making a transition to a different state, and a method for observing the agents. The purpose of the policy is to define how the agent maps and reacts to its observed environmental conditions by performing actions. The rewards are instantaneous numerical values the agent receives for performing a specific action when it is in a given state. The value function serves as the long time version of the reward function and its aim is to compute the discounted return from the current state to a terminal state that resulted from a specific policy choice. For a recent comprehensive review and introduction to reinforcement learning see Sutton and Barto (2018).

Deep reinforcement learning is a machine learning technique that combines deep learning and reinforcement learning concepts in order to learn an optimal agent’s behaviour based on its past experiences and actions, by iteratively evaluating the agent’s accumulated reward. Deep reinforcement learning is divided into three main categories which are value-based, policy-based and model-based approaches. Other reinforcement learning techniques include Monte Carlo, temporal difference and on-policy and off-policy methods. The objective of value-based methods is to allow an agent to learn a policy that maximizes its value function over a long sequence of actions. In policy-based approaches which can be either deterministic or stochastic, the goal of the agent is to learn a policy that results in the optimal value of the objective function. Model-based approaches require that a model of the environment is given to the agent or that the agent learns such a model in order to allow it to learn how to optimally perform tasks in that environment.

Q-Learning is a reinforcement learning method applied to solve path planning problems, but that does not require a model for an environment. Moreover, it is able to handle problems with stochastic transitions and rewards. The basic idea of Q-Learning is to find the optimal control policy by maximizing the expected total reward over all future steps. If a robot’s action *a* yields a real return *r*, then the objective is to obtain a strategy *Q*: *S* → *A* that maximizes these returns. Denote by *γ* ∈ [0, 1] a discount factor that weighs earlier rewards heavier then those obtained later, and by *δ* a map between the current state-action pair and the next state. The training is carried out based on the immediate return and the long term return of the action given by,

$$Q\left(s,a\right)=r\left(s,a\right)+\gamma \underset{a^{\prime }}{\max}Q\left(\delta \left(s,a\right),a^{\prime }\right)$$ (2)

The robot repeatedly observes the current state *s*, selects and executes a certain action *a*, observes the returned result *r* = *r*(*s*, *a*) and the new state *s*′ = *δ*(*s*, *a*). Any action *a* can be found then by solving,

$$a=\underset{a^{\prime }}{\mathrm{argmax}}\left[r\left(s,a\right)+Q\left(\delta \left(s,a\right),a^{\prime }\right)\right]$$ (3)

Deep reinforcement learning can assist in training robots to search unknown environments Li et al. (2019), Hu et al. (2020), and Peake et al. (2020) and allow it to learn to develop strategies that will be used to search and detect smart evaders without following carefully designed pre-planned trajectories or to assist searching robots to detect survivors in search and rescue missions. In Azar et al. (2021), a review on the usage of deep reinforcement learning techniques for drone applications is provided. In Zhang et al. (2021), a selective overview of theories and algorithms on multi-agent reinforcement learning is presented. In Oroojlooy and Hajinezhad (2022), a review on cooperative multi-agent deep reinforcement learning is given.

Since robots used for search and rescue missions are often used in environments that are at the very least partially unknown, perhaps due to an earthquake, an area devastated by a storm or a collapsing underground cavern, they need to have the ability to operate in changing environments for which they do not have a fully defined mathematically accurate model. This is also the case for UAVs participating in pursuit-evasion problems that take place in adversarial environments, for which complete information on the environment model and the opponent team is generally unknown. Hence, allowing autonomous agents to learn their path planning strategies by using reinforcement learning and deep learning techniques enables agents to iteratively learn how to operate in dynamically changing and partially known environments by exploring the best strategies that achieve their goal throughout the training process, leading to the application of these learned strategies in the field afterwards.

In Sun et al. (2021) multi-agent reinforcement learning for distributed cooperative targets search is considered. The authors develop a decentralized reinforcement learning method performed by UAVs in an area of interest. In the investigated scenario a team of UAVs cooperatively searches a partially known area with the objective of detecting an unknown number of targets. The team of searchers has access to an inaccurate prior distribution on the targets’ locations and it is assumed that both searchers and targets have limited kinematics and sensing capabilities, and that searchers have disk-shaped sensors. The paper leverages on advancements in reinforcement learning techniques and casts the problem of multi-agent cooperative search into a decentralized partially observable Markov decision process, showing its advantages over monotonic value function factorization (QMIX) Rashid et al. (2018) and random-based methods. The aim of the developed protocol is to find all targets in minimum time.

Multi-agent learning problems are usually solved by decomposing a complex multi-agent problem into a set of simple single-agent problems. In each of the single agent problems the environment is shared with that of the other agents. This method is referred to as independent Q-learning. Independent Q-learning achieves decentralization, a solution that is sought after in multi-agent problems since it allows to increase the number of agents without having to increase the computational burden, however it may result in agents that are unstable and continuously change their search policy. To lead the team to learn to decentrally coordinate their actions the authors propose to learn local Q-value functions for each agent. These functions are combined by using a mixing function and result in a joint action Q-value function. The selected mixing function represents the consistency between local and global chosen actions in the joint action value function of the searching team and in the individual value functions of the agents, which depend only the observations and situations the agent encountered.

In Wenhong et al. (2022), multi-target cooperative tracking guidance for UAV swarms using multi-agent reinforcement learning is investigated. The authors argue that current multi-agent reinforcement learning based methods rely on global information and are too computationally demanding to be used in large scale robotic swarms and hence propose a decentralized multi-agent reinforcement learning based method to address these limitations. The proposed method allows UAVs to learn cooperative tracking policies by using the maximal reciprocal reward of the swarm.

In Yue et al. (2019), a searching method for multiple dynamic targets using reinforcement learning scheme for multi-UAVs in unknown sea areas is proposed. The method creates a multi-UAV area search map is which includes models of the environment, UAV dynamics, target dynamics, and sensor detections. The constructed search map is updated and extended with a concept the authors refer to as territory awareness information map. Based on the created map and the constructed search efficiency function, a reinforcement learning method outputs a multi-UAV cooperative search path online.

In Vlahov et al. (2018), a UAV pursuit-evasion policy using reinforcement learning presents an approach to learn reactive maneuver policies for aerial engagement scenarios. The authors rely on deep A3C Mnih et al. (2016) algorithm which uses actor-critic networks. The actor-critic concept is based on using two separate networks. The actor-network estimates the optimal behavior, while the critic network estimates the reward of the behavior and uses rewards to train the actor. A3C or asynchronous advantage actor-critic is a model-free and on-policy algorithm operating in continuous action and state spaces. A3C uses one global actor-critic network together with multiple local actor-critic networks. Local networks rely on information obtained from the global network for initialization purposes, and reciprocally feed the global network by their continuously updated gradients. The authors learn using A3C an aerial combat behaviour learned in simulation and show its results on real-world flight tests highlighting the promise of using such techniques. An interesting extension to this work is to apply it in a multi-agent team against team setting.

In Niroui et al. (2019), a robot using deep reinforcement learning for search and rescue exploration in unknown cluttered environments is presented. The advantage of using reinforcement learning techniques in search and rescue missions is their ability to operate in challenging and dynamic environments containing obstacles. The work is the first one to apply deep learning based robot exploration in urban search and rescue environments and does so by combining frontier-based exploration with deep reinforcement learning that allows the robot to have obstacle avoidance capabilities and the ability to operate in unknown environments. In Frontier based exploration a robot is directed to explore the boundaries of an area that was already explored, hence it extends the frontiers and boundaries of the explored region.

In Hu et al. (2020), Voronoi-based multi-robot autonomous exploration in unknown environments *via* deep reinforcement learning is suggested. Autonomous exploration is a task in which a team of coordinated robots collaboratively explores an unknown area, and can be used in scenarios such as search and rescue and mapping and localization of unknown environments. The proposed approach reduces task completion time and energy consumption by designing a hierarchical control architecture that contains a high-level decision making layer together with a low-level target tracking layer. Cooperative exploration is achieved by using dynamic Voronoi partitions, which allows to reduce overlapping exploration sections through the partition of explored area between the exploring agents. A deep learning obstacle avoidance algorithm is used for collision avoidance with dynamic and previously unknown obstacles. The algorithm learns a control policy based on exploration of unknown areas by a human that guides and controls the robot team during the task, resulting in improved performance.

In Liu et al. (2021), cooperative exploration for multi-agent deep reinforcement learning is investigated. The authors identify an existing challenge in current multi-agent exploration methods which arises from the inability of agents to identify states worth exploring and the inability to coordinate exploration efforts that will lead agents to these states. In order to address this limitation, the authors propose that agents will share information about the goal between themselves in order to reach this goal in a coordinated manner by selecting the goal from multiple projected state spaces. The authors argue that previous multi-agent reinforcement learning approaches obtain good performance since they address the non-stationary issue of multi-agent reinforcement learning by using a centralized critic, however recent works prove the sub-optimality of these methods since they rely in classical exploration methods which use noise-based exploration which is a noisy version of the exploration policy implemented by the actor.

### 2.6 Task allocation in multi-agent search tasks

Multi-robot task allocation is concerned with distribution of tasks and goals between the members of the multi-robot team. Examples of tasks in multi-agent search tasks includes division and allocation of different areas to be searched, requiring robots to position themselves in certain locations in order to serve as a relay network to ensure communication connectivity between the team’s robots and division of tasks between heterogeneous robots of a team such as division of tasks between a ground and an aerial robot. Search and rescue tasks performed with multi-agent teams include collaborative mapping of unknown areas, situational assessment of affected areas, cooperative search of a designated area as well as cooperative area coverage.

After allocation of tasks to the individual robots is performed, each robot is responsible to implement its allocated task to the best of its ability. However, from the team’s perspective, some method of verification should be enforced to check if all robots performed their intended duties adequately and that no robot failed during the execution of the task. If a certain robot fails to complete its task, then the team must compensate for its member’s failure by sending additional robots to cover for the malfunctioned one by allocating tasks again or by updating the mission’s objective accordingly.

Generally, for search and rescue operations that often take place in harsh and challenging environments or for other search problems that take place in adversarial environments, implementing distributed algorithms for the robot team, provides some resiliency and robustness to malfunctioning agents.

In Gerkey and Matarić (2004), a formal analysis and taxonomy of task allocation in multi-robot systems is given. The study formulizes multi-robot task allocation problems using tools from optimization theory and operations research and specifically concentrates on methods for intentional robot cooperation through task-related communication and negotiation and a formal analysis of such methods.

In Mosteo and Montano (2010), a survey on multi-robot task allocation that focuses on service and field robotics is conducted highlighting the advantages and limitations of discussed approaches. The paper describes properties that are desirable in multi-agent task allocation which are decentralization scalability, fault tolerance, flexibility and responsiveness. The paper describes several solution models to the multi-robot task allocation problem which are categorized into centralized, stochastic, auction and behavioral models. The paper further identifies core aspects of task allocation strategies which are task decomposition, cost models, task models, execution models and task constraints.

In Korsah et al. (2013), a comprehensive taxonomy for multi-robot task allocation is provided. The survey specifically focuses on issues of interrelated utilities and constraints which were not addresses by previous surveys that focused more on independent tasks. The survey investigates and classifies multi-robot task allocation problems using tools and models from combinatorial optimization and operations research.

In Liu and Nejat (2016), multi-robot cooperative learning for semi-autonomous control in urban search and rescue applications is explored. In urban search and rescue tasks it is essential for robots to coordinate task allocation and execution in order to minimize search time and maximize the number of detected survivors. A multi-robot cooperative learning approach for a hierarchical reinforcement learning based semi-autonomous control architecture is developed to allow a team of robots to cooperatively learn how to explore and how to detect survivors in challenged urban search and rescue settings. Effective task allocation between the team of searchers is learned alongside efficient execution of the allocated tasks. Furthermore, the developed approach allows human-robot interaction by enabling a human operator to intervene when robots request to hand over the task to humans since they determine that they cannot perform it in a satisfactory manner by themselves. Therefore, robots take cooperative decisions on task allocation between the team members and between robots and humans by assessing their performance on their designated tasks.

In Nunes et al. (2017), a taxonomy for task allocation problems that focuses on temporal and ordering constraints is provided. In Ferri et al. (2017), an overview on cooperative robotic networks for underwater surveillance is provided, with a specific focus on multi-robot task allocation for underwater multi-robot surveillance settings.

In Sung et al. (2020), distributed assignment with limited communication for multi-robot multi-target tracking is investigated. Tracking of moving targets is performed by employing a team of mobile robots that can choose to execute their actions based on a set of motion primitives with an objective of maximizing the total number of tracked targets by the team or its tracking quality. Emphasis is put on limited communication setting and short broadcast time. Hence, these considerations naturally fit a distributed setting and algorithms that achieve the team’s goals in finite time and adhere to the communications constraints are developed. Two algorithms are proposed. The first is a greedy algorithm that achieves twice the optimal centralized performance and requires a number of communication rounds between robots that is equal to at most the number of robots. The second algorithm allows the designer of the tracking team to trade off tracking quality and communication time by selecting the value of two tuning parameters.

In Rodríguez et al. (2020), wilderness search and rescue with heterogeneous ground and aerial multi-robot systems is explored. In this work the authors argue that using a team of heterogeneous robots improves the robustness and efficiency of the team compared to using a team of homogenous agents. A multi-robot task allocation algorithm deployed on ground and aerial vehicles that uses the market-based approach to optimize the mission resources is proposed. The algorithm considers the availability of vehicles, task’s characteristics and payload requirements and in turn generates a plan of tasks ans charging commands for each vehicle.

In Carrillo et al. (2021), communication-aware multi-agent metareasoning for decentralized task allocation is considered. Multi-agent metareasoning refers to the ability of agents to reason about their decision-making. The presented algorithm develops a policy that allows a multi-agent team to choose between several task allocation possibilities depending on the communication quality. Due to the decentralized nature of the algorithm and because all agents run the same selection policy, some or all of the team‘s agents jointly switch between task allocation algorithms based on the quality of the communication level they sense. Reactive synthesis is used to generate the policy based on high-level specifications written in Linear Temporal Logic that enable to encode the agents’ switching behavior based on communication level they observe.

In Amir et al. (2022), multi-agent distributed and decentralized geometric task allocation is explored for swarms of agents. The agents are assumed to be very simple and are oblivious, identical, have a limited sensing range and cannot explicitly communicate with each other. The assumptions in this work is that time is discrete and that tasks are represented by an a-priori unknown demand profile that dictates how many agents are required to be in each location in order to complete their allocated task. The paper assumes a stricter limited visibility and purely-local information setting compared to previous works that considered target assignment and task allocation for a decentralized swarm of agents that aims to configure itself in the explored environment for the purpose of achieving a desired spatial distribution, such as the works by Cortes et al. (2004), Schwager et al. (2009), and Schwager et al. (2011).

Two task allocation problems are investigated: signal coverage and target assignment. Signal coverage seeks to distribute robots in the environment so that they sufficiently cover an a-priori unknown location dependent and potentially time-varying profile function with signals the agents emit. The goal of the agents performing this task is that their combined emitted signals approximate the desired demand profile function. Target assignment aims to allocate the swarm’s agents in order to detect targets that are placed in discrete and unknown locations by having the searching agents move to the targets’ locations. This is achieved by setting highly concentrated environmental signals at the location of each target, resulting in the organization of the swarm based on the needs of the tasks. Interestingly, in the investigated target assignment scheme, different targets require different numbers of agents in their vicinity in order to be detected, simulating the fact that based on the complexity of different tasks, they require a different number of agents to complete them successfully.

The autonomous swarm agents’ goal is to configure themselves according to the demand function by using only local information available to them through the demand function, and by considering only the locations of other agents they sense in their local neighborhood. The task allocation problem is solved by using an optimization-based approach resulting in attraction and repulsion forces applied to the exploring agents, repelling them from each other in order to explore more of the area and drawing them near targets they need to detect. The results of the paper demonstrate that despite the strict assumptions about the individual agent’s capabilities, a large variety of challenging tasks can be solved by equipping a sufficiently large swarm of simple agents with the proposed local attraction and repulsion dynamics.

### 2.7 Cooperative sensing in multi-agent search tasks

Cooperative sensing for a team of robots, enables robots to share and obtain vital information from peers or from the infrastructure. This allows to treat the searching multi-robot team as a connected team with extensive knowledge and understanding of its environment. Better informed decisions could be made, as autonomous robot cooperation is expected to improve individual local sensing capabilities. This in turn can lead to more accurate perception, detection and tracking capabilities of the robot team, thus allowing it to perfect its search plan and its ability to succeed in its planned mission. In order to enable cooperative sensing and allow connected robot teams to realize their full potential, advancements need to be performed both in the communication, perception and planning domains. We list below several tasks that could benefit from cooperative multi-agent sensing.

An additional important aspect worth considering in multi-robot perception is the reliance of state-of-the-art perception algorithms, especially in the computer-vision domain, on deep learning based approaches. While state-of-the-art results are achieved using such methods across essentially all visual perception domains, such as object detection, segmentation, tracking, classification and many more, these methods require high computational power and dedicated hardware such as graphical processing units (GPUs) which consume a significant amount of a robot’s battery and increase the weight of its payload considerably Howard et al. (2017), Hadidi et al. (2018), and Kumar et al. (2020). Therefore, there is a need to perform both hardware and software optimizations and adaptions for deep learning based algorithms such as developing more computationally light models to allow their specific usage for mobile robotic applications, especially for their usage in drones which are limited the most by these constraints.

In Queralta et al. (2020) several challenges regarding fusion between different data sources in multi-agent perception are listed. These challenges occur since the agents are located at different locations, different lighting conditions, can have different sensors and move with respect to one another. Among the challenges that need to be addressed are where to perform data fusion, how to determine if agents are observing the same landmarks and how to decide which observations provide more information and are more beneficial to the knowledge of the robot team.

The paper further describes a classification to 4 categories present in multi-agent target tracking which are cooperative tracking, cooperative multi-robot observation of multiple moving targets, cooperative search, acquisition, and tracking where the last category is multi-agent pursuit evasion. Cooperative tracking seeks to track moving objects using several robots that share information. Cooperative multi-robot observation of multiple moving targets seeks to increase the total time in which all targets are observed. Cooperative search, acquisition, and tracking constantly switches between searching and tracking of moving targets. For a detailed review on the perception aspect of multi-agent pursuit evasion observation and tracking of multiple targets and on how it relates to control techniques for cooperative mobile robots that need to solve such challenges see Khan et al. (2016).

In Song et al. (2008), a method for multi-robot cooperative sensing and localization is developed. Cooperative localization is achieved by fusing sensory data obtained through each robot’s visual detections that aim at identifying other robots and localization itself. In Banfi et al. (2015), investigates multi-target tracking in cooperative multi-robot systems. In this work, a team of autonomous robots with limited range sensors must observe a set of mobile targets. The team cooperatively plans its motions to maximize the time that each target will be inside the sensing range of at least a single robot.

In Zhou and Tokekar (2018), an algorithm for active target tracking with self-triggered communications in multi-robot teams is proposed. The team of robots moves on the boundary of the environment and seeks to be in a formation that enables the robots to optimally track a target that moves in the interior of the environment. The robots can measure distances to the target. The objective is to reduce the amount of shared information throughout the process of converging to the optimal configuration, and this is achieved by developing a communication protocol that informs robots when to exchange information with their neighbors and when it is safe to operate with possibly outdated information.

In Scherer and Rinner (2020), an algorithm for multi-UAV surveillance with minimum information idleness and latency constraints is developed. There are two goals for the UAV team, which it attempts to achieve by using cooperative data transport. The first is to minimize the information idleness, defined as the lag between the start of the mission and the time at which data captured at a sensing location arrives at the base station. The second goal is to minimize the latency which is defined as the lag between capturing data at a sensing location and its arrival at the base station.

#### 2.7.1 Cooperative localization

Cooperative localization of robots may open the door to deployment of multi-robot teams in GPS or GNSS denied areas. These scenarios may occur in the context of multi-agent search tasks in pursuit-evasion domains where navigation signals are deliberately blocked, jammed or spoofed or in environments where the signal received from satellites is seriously degraded and poor, thus requiring robots to localize themselves without relying on such technologies. These scenarios occur in search and rescue operations in mediums such as underwater, underground and even in indoor settings, important domains that must be addressed differently than open-air scenarios.

In order to generate a more accurate representation of a robot’s surroundings, sensory information from robots in the vicinity and information from sensors that may be embedded in the infrastructure should be combined. Since measurements always contain some degree of noise, algorithms that fuse measurements from different sources mitigate this effect and reduce perception uncertainty. Likewise, access to information that is not available to a certain robot and could be acquired from its neighbors, improves the individual robot’s performance.

Works on this topic appears in a different context in literature as part of the vision for a connected vehicle environment envisioned for the deployment of autonomous vehicles. We believe that similar concepts may be used in other robotic tasks where sharing of information between team members can improve results. A map merging approach that aligns multiple local sensing maps to make observations consistent with each other, is proposed in Kim et al. (2014). Frequent transmissions of a local sensing map are computationally inefficient and bandwidth consuming. Hence, the authors choose to explore a more efficient approach by automatic alignment which is gained once a vehicle is localized on the global map. Cooperative localization is also possible through the exploitation of correlations in joint and relative observations, such as relative range and relative bearing which are also applicable in multi-robot search tasks carried out by drones which often use these types of measurements in their algorithms.

In Gao et al. (2019) a detailed review on theory, research, and practice of cooperative localization and navigation is provided. In Zhu and Kia (2019), cooperative localization under limited connectivity is investigated. The authors present 2 decentralized multi-agent cooperative localization algorithms where inter-agent state estimate correlations are accounted for implicitly for the purpose of reducing the communication cost. Individual agents localize themselves in a global coordinate frame with a local filter, by using sporadically absolute measurements and through the correction of their pose estimate when they receive a relative measurement to another agent. Since agents correct their pose only when they obtain a relative measurement from another agent, no global connectivity preservation between robots is required.

In Paull et al. (2013), a review on Autonomous Underwater Vehicle (AUV) navigation and localization is provided. The underwater medium is a challenging one to navigate through since GPS signals cannot penetrate to the depths of body of water due to their attenuation. While traditional approaches used inertial sensors or underwater beacons the accuracy of these approaches is limited. In recent years, due to the advancement of simultaneous localization and mapping (SLAM) techniques underwater localization accuracy improved dramatically, and the paper investigates these advancements. In Ebadi et al. (2022) a recent survey that investigates the current state and future research directions of SLAM in challenging underground and subterranean environments is provided.

##### 2.7.1.1 Minimal sensor configuration

The problem of multi-robot cooperative localization where each robot is equipped with a designated sensor that is sufficient for it to complete its intended task had been previously investigated. However, if robots were to operate in a connected environment under a shared sensory information paradigm, the individual sensing capabilities of each robot may be reduced. In the context of multi-agent search tasks this translates to the ability of a robot to carry a lighter payload, consume less energy and thus allow it to increase the duration of the mission it executes. This in turn may lead that a certain mission can be executed faster and with less and simpler robots. This research question was investigated in the context of cooperative and connected vehicles in Shen et al. (2016), in a work that set qualitative and quantitative sensor requirements that allow vehicles to cooperatively localize themselves up to an acceptable error. The work tackles the cooperative localization of a distributed set of vehicles. The authors design a minimal and scalable sensor configuration which allows cooperative localization of a vehicle fleet on an urban road. Drawing inspiration from such works may be beneficial to the advancement of multi-robot search tasks as well.

#### 2.7.2 Multi-agent active perception

Multi-agent active perception methods aim to adapt the behaviours of agents for the purpose of receiving more beneficial perception inputs from the agents’ sensors. This implies that the robot team understands the goal of its mission and knows what it means to obtain more beneficial inputs and how it can plan its movements and use its available resources to achieve it.

In search and rescue operation active perception is used in various tasks such as search for victims, path finding in challenging environments, obstacle avoidance and target detection and tracking. Using active perception in these tasks enables the robot team to have much needed flexibility in adapting its behaviour to the changing conditions of its mission Queralta et al. (2020).

Multi-agent teams that use active perception algorithms perform alongside their main objectives specific movements and apply reasoning that allows them to improve their performance by acquiring better data through the selection of the next set of actions that need to be performed by the team to improve the results of its main task. This consideration presents a challenge in training data acquisition since there is a large number of possible agents’ movements to be performed and how to choose this set of actions in the data collection phase is not a trivial question. Additionally, there is need to define the notion of a good action and tie it to the observation received after performing the action, which is challenging. Usage of simulation in both data collection and training phases calls for sim-2-real techniques to be applied since there is a large pool of potential actions agents can choose from and this complicates the data collection of such methods with real robots. Currently, the most active research direction in active perception is reinforcement learning which inherently has the notions of rewards received when an agent takes a specific action and therefore it is very suitable for active perception tasks.

Active perception is a useful tool that is naturally applicable for multi-agent search and rescue missions. In Acevedo et al. (2020), a cooperative multi-robot search algorithm that combines usage of a particle filter and active perception is presented. Collaborative search performance is optimized by actively maximizing information collected by robots throughout their search. The algorithms assumes a certain degree of uncertainty in the data, and hence uses the particle filter for active collaborative perception, yielding dynamic distribution of robots in the explored area.

In Ahmad et al. (2013), cooperative multi-agent tracking and active perception formation control techniques are combined in order to maximize the tracking performance of a target by using a non-linear model predictive control algorithm. In Tallamraju et al. (2019), an additional work that uses active perception techniques, model predictive control and formation control for multi-agent tracking is presented. Define the tracking error as the distance between the robots and the target and the formation error as the difference between the bearing angels of the robots and the tracked target. Model predictive control is obtained through the decoupling of the tracking and formation errors and enables real-time computations of safe UAV trajectories throughout the tracking process while surrounding the target with a desired UAV formation. The estimation errors are reduced by optimizing the locations of searchers to be in a relative position to the target which will be as close as possible to the center of the field-of-view of each robot, thus allowing for better collaborative detection and tracking capabilities of the team.

#### 2.7.3 Motion coordination

Sharing information on a planned robot’s trajectory should help cooperative robots that perform search tasks in challenging dynamic domains to predict dynamic changes in the environment more accurately. If trajectories overlap or violate safety requirements, conflict detection and resolution algorithms must be applied. One type of distributed conflict resolution mechanism is introduced in Lin et al. (2017) in the context of connected vehicles. The conflict resolution algorithm for each vehicle is decoupled temporally and allows connected vehicles to navigate safely and efficiently through intersections. A vehicle computes the desired time slots to pass the conflict zone by solving a conflict graph locally based on the information that was broadcast from other vehicles. In the motion planner part, a vehicle computes the desired speed profile by solving a constrained optimization problem. The paper provides theoretical guarantees, that the combination of local vehicle decisions solves the conflicts globally. Such solutions are applicable also in the context of general multi-robotic tasks where a team of robots operates in a challenging, unknown and dynamic environment and may assist in providing solutions for the particular case of challenging pursuit-evader search tasks.

#### 2.7.4 Multi-agent active search

Multi-agent active search is an active learning problem whose goal is to locate a set of targets in an unknown environment by allowing a searching team of robots to actively perform sensing actions that depend on all past observations of the team by coordinating the movements of searchers to actively make informed data-collection decisions. Active learning is a branch of machine learning focused on learning algorithms that can interactively query an information source to label new data points with desired outputs. The practical need of developing an ability for teams of UAVs to collaborate safely in search tasks in a shared environment is one of the major driving forces for advancements in multi-agent active search.

In Igoe et al. (2021) multi-agent active search is investigated from the perspective of reinforcement learning. The authors argue that the main reasons that recent approaches are limited are since they are myopic in a sense that they do not optimize the information gain throughout the whole trajectory of search, but rather for limited time horizons, or that they introduce strong biases that degrades the performance. An additional limitation that is highlighted is the computational burden of current approaches, that are not scalable to large multi-agent teams without prior optimization for a pre-defined team configuration, and thus makes their application infeasible in real-world missions. Additionally, it is mentioned that since most prevailing multi-agent active search approaches are based on rather general strategies to obtain a wide dispersal of agents, and not on algorithms that incorporate information on the particular investigated search scenario this leads to sub-optimal decisions. By using a deep reinforcement learning based approach, the authors are able to both reduce computation time and optimise for non-myopic objectives by rewarding behaviour that results in good performance for the entire trajectory of the searching team.

### 2.8 Open research questions

In this section we list several interesting research questions regarding the topics concerning multi agent search systems discussed which should result in promising results that will enable their advancement and application in real-world settings.

Future work on guaranteed smart opponent detection can be performed on several fronts. The first is developing guaranteed detection strategies for agents having a sensor model that models actual visual sensors. Such an analysis provides a generalization to the sensors used in previous works. Usage of such sensors with a carefully designed search protocol that utilizes team cooperation by using pairs of sweepers implementing pincer sweeps and equipped should provide results that are both theoretically optimal and applicable in real-world scenarios.

Another promising research direction should consider discrete search for smart targets using pincer strategies. Contrary to the topics discussed in previous chapters, in this topic, the environment over which the search is performed will be discrete (e.g., a regular grid environment or a general planar graph), and the mission of the searching team of robots will be to locate the smart opponents. In such environments, preliminary work indicated that dealing with obstacles is quite straight forward. Extension of the established results and methods from the continuous to the discrete domain will enable the usage of the results in many other applications.

An additional extension is application of pincer-based search protocols in challenging environments containing dynamic obstacles. This shall be achieved by combining pincer-based methods performed by sub-teams of sweepers together with multi-agent deep reinforcement learning techniques that enable agents to operate in unknown environments. Furthermore, another important topic to consider is online formation of sweeping sub-teams and their application in search tasks for smart evaders and invaders. This will allow usage of the established results in more general environments and enable the multi-agent searching team to handle cases where the team needs to reconfigure itself in order to detect all evaders or when other agents need to replace a malfunctioning searcher.

Deep reinforcement learning can greatly benefit multi-agent search applications since it allows agents to operate in unknown environments and environments that do not have a defined mathematical model and cope with uncertainties in the mission Azar et al. (2021). Topics such as operation of large numbers of searching robots in a shared environment, especially drones, can be improved by developing collaborative obstacle avoidance algorithms that rely on local communication between robots and are verified to be safe. Additionally, since many of the existing works rely on data from simulation it is important to advance sim-2-real techniques Queralta et al. (2020) that will enable to apply theoretical and stimulative work in the field as well. Furthermore, decentralized online reinforcement learning based path-planning algorithms that rely only on the agent’s sensory inputs and on inputs of its local neighbors will allow to alleviate the heavy need to rely on GPS, thus allowing application of search and rescue robotic teams in areas with low or no GPS signal, in indoor environments or environments in which GPS signal is blocked. Furthermore, such methods will also provide resiliency to the robotic team against cyber attacks Gil et al. (2017), Zhou and Tokekar (2021), and Cavorsi and Gil (2022). Additionally, robust deep multi-agent reinforcement learning techniques should be developed to enable handling adversarial opponents that may wish to degrade the team’s performance, especially in surveillance and pursuit-evasion scenarios. Developing algorithms for generation of energy efficient trajectories is also an interesting research direction that will promote drone based search systems García-Martín et al. (2019).

In the topics of run time and optimization of machine learning based perception algorithms for robotic applications, more effort should be put on developing computationally light algorithms that are more suitable for deployment on robots with critical energy limitations such as drones Bouguettaya et al. (2019). Future research on collaborative multi-robot multi-human teams Chen and Barnes (2014) should consider building trust between human operators and semi-autonomous robotic search teams. A possible path to achieve such trust is by providing theoretical guarantees on the performance of robotic systems in real-world tasks and by developing suitable verification methods for the safety of their operation in performing rescue missions Rahman et al. (2021). An additional interesting research direction is to develop online risk bounded motion planning algorithms for multi-agent robotic teams that operate in a shared environment with human search and rescue personnel.

Future research in reconfigurable team formations for search and surveillance applications Cao et al. (2012) and Cortés and Egerstedt (2017) should consider developing more decentralized approaches that are suitable for robotic teams with a large number of searchers that do not rely on extensive sharing of information between the team’s members. Furthermore, work on connectivity preserving formations in exploration of unknown areas such as underwater Ferri et al. (2017) or underground environments Rouček et al. (2020) and Ohradzansky et al. (2021) will surely enable deployment of such forms in real-world search missions. Research on reconfigurable team formations can also assist in situations where a robot malfunctions Oh et al. (2015) and other robots need to determine how they should reconfigure themselves in order to compensate for the robot’s failure and achieve the best team performance. Additionally, development of algorithms that enable adaptive behaviour and reconfigurable formations of robotic teams can assist in multi-agent pursuit-evasion problems, especially in unknown and dynamically changing environments.

In the topics of cooperative sensing for multi-agent search tasks, further research should be conducted in the topics of minimal sensor configuration that allows the robot team to perform its task. Advancing research in this topic will enable drone teams to execute longer missions, an essential need in search and rescue missions, by carrying only the necessary sensor suit needed for the mission, based on the knowledge that it can utilize information obtained from other agents as well. Similarly, cooperative localization can also benefit from sharing of sensory inputs from other members of the team allowing to reduce computational power and sensor requirements as well Paull et al. (2013) and Zhu and Kia (2019).

In the topic of task assignment for multi-agent searching systems further research should be performed in online task assignment and on task assignment for heterogeneous robot teams Rizk et al. (2019). It is also worth to investigate decentralized dynamic task allocation algorithms Nunes et al. (2017) that allow to perform the task assignment on the local level or on sub-teams. This will open the door to the deployment of multi-agent robotic teams with larger numbers of participating robots by allowing computationally feasible calculations that do not require heavy optimization to be solved.

## 3 Conclusion

In this work we provide a current view on the multiple topics related to multi-agent search. We describe recent works in probabilistic and guaranteed search protocols for smart opponent detection. Afterwards, we investigate additional research trends that are relevant to multi-agent search including reconfiguration of multi agent teams, usage of reinforcement learning for multi agent search applications, multi-agent active search, task assignment for multi-agent search problems, cooperative sensing for multi-agent search and practical considerations in the design of multi-agent search systems. We later provide future research directions that we deem are important for further investigation in order to promote the advancement of the multi robot search systems of the future.
